# Comparison of outcomes of self-expanding versus balloon-expandable valves for transcatheter aortic valve replacement: a meta-analysis of randomized and propensity-matched studies

**DOI:** 10.1186/s12872-023-03397-3

**Published:** 2023-07-31

**Authors:** Baiqiang Wang, Zeyuan Mei, Xiao Ge, Yunyi Li, Quan Zhou, Xiao Meng, Guipeng An

**Affiliations:** National Key Laboratory for Innovation and Transformation of Luobing Theory; The Key Laboratory of Cardiovascular Remodeling and Function Research, Chinese Ministry of Education, Chinese National Health Commission and Chinese Academy of Medical Sciences, Qilu Hospital of Shandong University, Jinan, China

**Keywords:** Transcatheter aortic valve replacement, Self-expanding valves, Balloon-expandable valves, New generation valves, Meta-analysis

## Abstract

**Background:**

The postoperative outcomes of transcatheter aortic valve replacement (TAVR) with the new generation of self-expanding valves (SEV) and balloon-expandable valves (BEV) remain uncertain.

**Methods:**

We conducted a meta-analysis based on randomized controlled trials (RCTs) and propensity score-matched (PSM) studies to evaluate the performance of the new generation TAVR devices, with a focus on Edwards SAPIEN 3/Ultra BEV, Medtronic Evolut R/PRO SEV, and Boston ACURATE neo SEV. Our primary endpoints were mortality and complications at both 30 days and one year post-operation.

**Results:**

A total of 4 RCTs and 14 PSM studies were included. Our findings showed no significant difference between SEV and BEV regarding 30-day and 1-year mortality rates. ACURATE SEV required less permanent pacemaker implantation (PPI) at 30-day as compared to SAPIEN BEV, while Evolut SEV required a higher rate of PPI than SAPIEN BEV. The incidence of stroke, major or life-threatening bleeding (MLTB), major vascular complications (MVC), coronary artery obstruction (CAO) and acute kidney injury (AKI) did not differ significantly between the two groups. SEV had a larger effective orifice area (EOA) and lower mean transvalvular gradients (MPG) compared to BEV. However, there was an increased risk of paravalvular leakage (PVL) associated with SEV.

**Conclusions:**

In terms of 30-day mortality, stroke, bleeding, MVC, AKI, CAO, and one-year mortality, there was comparability between the two valve types following TAVR. SEV was associated with better hemodynamic outcomes, except for a higher incidence of PVL. Compared to SAPIEN BEV, ACURATE SEV had a lower risk of PPI at 30 days, while Evolut SEV was associated with a higher risk of PPI. These findings underscore the importance of personalized valve selection.

**Supplementary Information:**

The online version contains supplementary material available at 10.1186/s12872-023-03397-3.

## Introduction

In patients with symptomatic aortic stenosis ranging from low to high risk, transcatheter aortic valve replacement (TAVR) has emerged as an effective alternative to surgical aortic valve replacement since its introduction by Cribier in 2002 [1–3]. There are two commercially available types of transcatheter heart valves (THV): balloon-expandable valves (BEV) and self-expanding valves (SEV). A BEV utilizes the radial strength of the accompanying balloon to facilitate expansion. In contrast, an SEV automatically deploys and expands until it encounters the resistance of the annular wall, thereby adapting to the anatomical characteristics of the aortic annulus [4].

In recent years, both platforms have undergone significant modifications aimed at enhancing the safety and effectiveness of the procedure. Currently, the most commonly used balloon-expandable valve (BEV) in clinical practice is the SAPIEN 3/Ultra BEV (Edwards Lifesciences Corporation, Irvine, CA, USA). The commonly used self-expanding valves (SEV) include the Evolut R/PRO SEV (Medtronic Inc., Minneapolis, MN, USA) and the ACURATE neo SEV (Boston Scientific, MA, USA). While these two valve platforms share several features in terms of valve design and procedural characteristics, they also exhibit differences in other aspects. The SAPIEN 3/Ultra is BE intra-annular trileaflet bovine pericardium valves mounted on a cobalt-chromium frame. The Evolut R/PRO is a SE supra-annular trileaflet porcine pericardium valve mounted on a nitinol frame. The ACURATE neo is a SE supra-annular trileaflet porcine pericardium valve mounted on a nitinol frame. In contrast to Evolut SEV, the ACURATE neo features a unique, top-down two-step deployment mechanism [5].

Prior studies have mostly focused on comparing first-generation Corevalve or SAPIEN XT devices [6, 7], and limited data are available regarding the comparison between current iterations of BEV and SEV. Therefore, in this meta-analysis, we evaluated and compared the postoperative outcomes of patients with symptomatic severe aortic stenosis treated with TAVR using the new-generation BEV and SEV. Most studies comparing the two THV types are observational studies, with only a few randomized controlled trials available. Hence, we included only randomized and PSM studies to ensure the reliability of our meta-analysis.

## Methods

We adhered to the PRISMA (Preferred Reporting Items for Systematic reviews and Meta-Analyses) statement for systematic reviews and meta-analyses when designing this study [8]. Our literature search encompassed PubMed, EMBASE, and Cochrane library from their inception through February 12, 2023. The search strategy utilized the following keywords and Medical Subject Headings (MeSH): “transcatheter aortic valve replacement,” “transcatheter aortic valve implantation,” “TAVR,” “TAVI,” “self-expanding valves,” and “SEV.” In addition, we reviewed the references of all identified studies for any other potentially relevant publications. The details of our search strategy are presented in Supplementary Table 1. Two independent searchers (X.G. and Z.M.) conducted the literature searches, and any issues were resolved through discussion or consultation with a third searcher (Y.L.) if needed.

To determine whether the studies will ultimately be used in the research, two reviewers independently screened the title and abstract of each reference. In the screening process, Endnote 20 document management software will be used. The following criteria were used to determine study inclusion: (1) patients undergoing TAVR for aortic stenosis; (2) trials comparing new generation SEV with the new generation BEV; (3) only RCT and PSM studies were considered. The primary outcomes of interest were mortality and complications. Several complication outcomes were of interest, including stroke, permanent pacemaker implantation (PPI), major or life-threatening bleeding (MLTB), major vascular complications (MVC), acute kidney injury (AKI), coronary artery obstruction (CAO), mild paravalvular leak (PVL), moderate to severe PVL, mean transvalvular gradients (MPG), and effective orifice area (EOA).

**Fig. 1:**
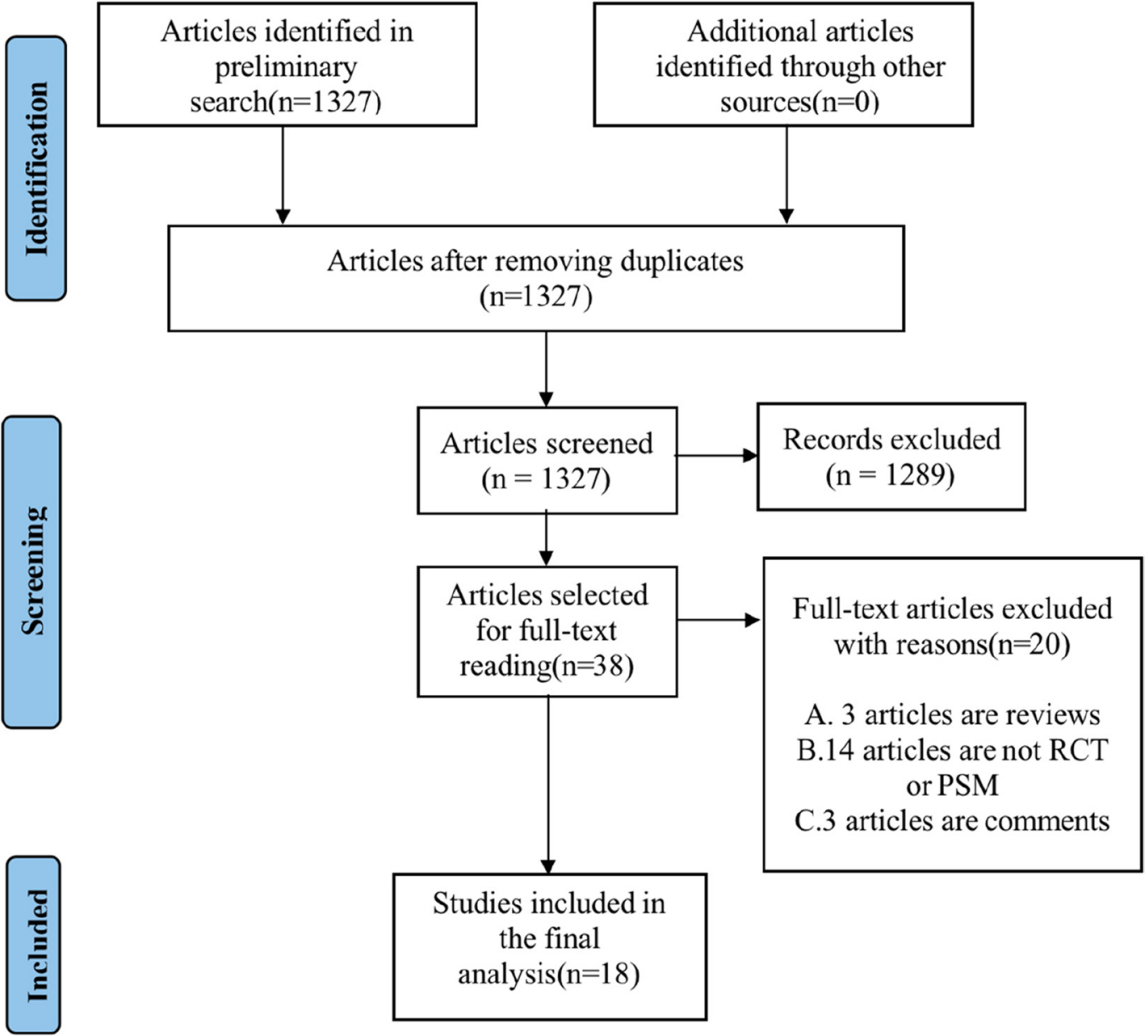
The flow diagram for study search process

Two reviewers (Z.M. and Y.L.) independently collected the following data from the text, tables, and figures: (1) characteristics of the included studies; (2) baseline characteristics of the study population; and (3) outcomes of interest. A pre-specified form was used for data extraction. The risk of bias of RCT was assessed using the Cochrane Collaboration's risk of bias tool [9], and the quality of PSM studies was evaluated using the Newcastle–Ottawa Scale [10]. The results of the quality assessments are presented in Supplementary Table 2 and Table 3.

Categorical variables are presented as percentages and continuous variables as means and standard deviations (SD). Some studies reported median and interquartile range, which were converted to mean and SD; median is considered as mean, and SD is calculated by dividing the interquartile range by 1.35. Review Manager (RevMan, Version 5.4, The Cochrane Collaboration, 2020) was used for meta-analysis. Based on the Mantel–Haenszel method with random-effects models, forest plot outcomes were expressed as risk ratios (RRs) and 95% confidence intervals (CIs). The heterogeneity of outcomes between the studies was calculated by I^2^. We calculated the I^2^ statistic and degree of freedom (df) to estimate variation between studies. We defined an I^2^ < 25%, 25%—50%, and > 50% as low, moderate, and high heterogeneity, respectively. Inverse variance was used to calculate pooled mean differences (MDs) with 95% CIs for continuous variables. Hypothesis testing was conducted at the level of 0.05 to ensure statistical significance.

Subgroup analysis was performed based on the design type of the study, and the results are presented in Supplementary Fig. 1. To test for robustness and explore heterogeneity in the pooled results, sensitivity analysis was conducted by removing one or more specific studies from the collection at a time. Funnel plots were generated for analyses with > 10 studies and were visually inspected for the asymmetrical distribution of data points across the vertical treatment effect axis. We used the STATA statistical software package (Version 16.1, StataCorp, College Station, Texas,USA) to create funnel plots (Supplementary Fig. 2) and perform Egger statistics.

## Results

**Fig. 2:**
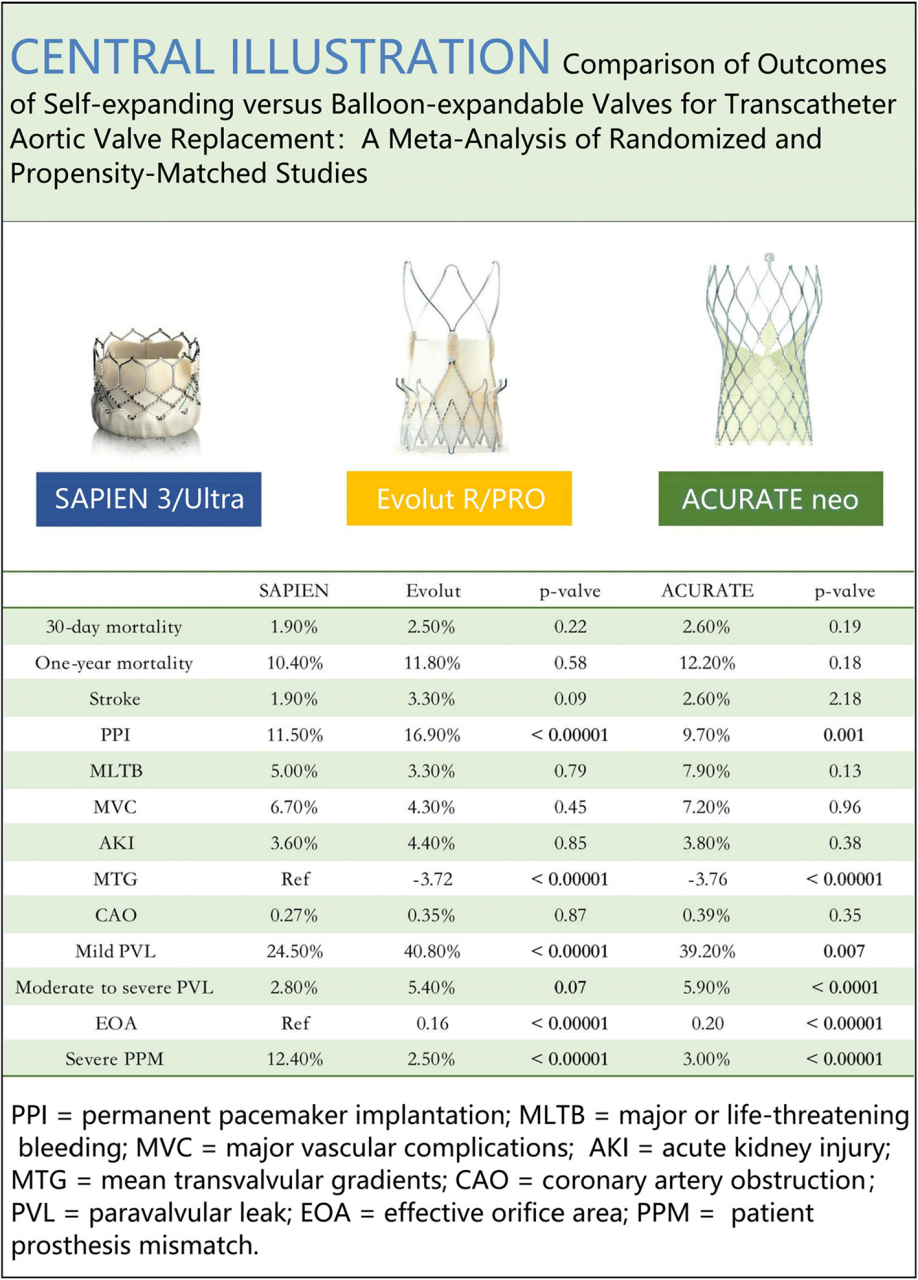
Clinical and echocardiographic outcomes after transcatheter aortic valve replacement according to Self-expanding (Evolut R/PRO, ACURATE neo) versus Balloon-expandable (SAPIEN 3/Ultra) Valves

Out of the 1327 articles initially found, 1289 were deemed irrelevant and excluded based on the titles and abstracts. The full text of the remaining 38 records was carefully reviewed. One study conducted by Deharo et al. was excluded from the meta-analysis due to its potential bias issues as it included a large cohort of tens of thousands of individuals [11]. Finally, a total of 18 publications were included in the analysis [12–29]. The details of the literature search and screening processes are presented in Fig. 1. Lanz et al. and Kim et al. reported the early and long-term outcomes of the SCOPEI RCT. Thiele et al. and Feistritzer et al. reported the early and long-term results of the SOLVE-TAVI RCT, respectively. Additionally, van Nieuwkerk et al. and Vlastra et al. reported early and long-term outcomes from the CENTER trial. The remaining 12 articles were observational studies based on propensity score matching, and a total of 9641 patients were included, with 4678 treated with SEV-TAVR and 4963 with BEV-TAVR. The main results of our study were presented in Fig. 2. The study and patient characteristics are summarized in Table 1** and **Table 2, respectively.

**Table 1:** The characteristics of the studies

Study	Year	Valve type	Trial	Sample size SEV/BEV	Follow-up	Design	Center	NOS/Bias risk
**Barth**	2019	ACURATE neo SAPIEN 3	-	329/329	319 ± 291 days	PSM	multicenter	9
**Costa**	2018	ACURATE neo SAPIEN 3 Evolut R	-	96/48	1 year	PSM	single center	9
**Costa**	2022	Evolut PRO SAPIEN 3 Ultra	OPERA-TAVI	683/683	30 days	PSM	multicenter	9
**Finkelstein**	2018	Evolut R SAPIEN 3	-	126/126	3 years	PSM	multicenter	8
**Hase**	2020	Evolut R SAPIEN 3	OCEAN-TAVI	69/69	1 year	PSM	multicenter	9
**Husser**	2017	ACURATE neo SAPIEN 3	-	311/622	30 days	PSM	multicenter	8
**Lanz**	2019	ACURATE neo SAPIEN 3	SCOPEI	372/363	30 days	RCT	multicenter	Low risk
**Kim**	2021	ACURATE neo SAPIEN 3	SCOPEI	372/363	1 year	RCT	multicenter	Low risk
**Mangieri**	2020	Evolut R/PRO SAPIEN 3	BEAT	77/77	1 year	PSM	multicenter	9
**Mauri**	2017	ACURATE neo SAPIEN 3	-	92/92	1 year	PSM	multicenter	9
**Pellegrini**	2023	ACURATE neo 2 SAPIEN 3 Ultra	-	472/472	30 days	PSM	multicenter	9
**Potratz**	2022	Evolut Pro SAPIEN 3	-	170/170	30 days	PSM	multicenter	9
**Rheude**	2022	Evolut R/PRO SAPIEN 3 Ultra	-	467/467	30 days	PSM	multicenter	9
**Schaefer**	2017	ACURATE neo SAPIEN 3	-	104/104	30 days	PSM	single center	8
**Thiele**	2021	Evolut R SAPIEN 3	SOLVE-TAVI	219/219	30 days	RCT	multicenter	Low risk
**Feistritzer**	2020	Evolut R SAPIEN 3	SOLVE-TAVI	219/219	1 year	RCT	multicenter	Low risk
**van Nieuwkerk**	2021	Evolut R SAPIEN 3	CENTER	791/614	1 year	PSM	multicenter	8
**Vlastra**	2018	Evolut R SAPIEN 3	CENTER	1091/1122	30 days	PSM	multicenter	8

*PSM* Propensity score matching, *RCT* Randomized controlled trial, *NOS* Newcastle–Ottawa Scale

**Table 2:** The baseline characteristics of the patients

Study	Study period	Valve type	Age, years	Male, %	STS risk score, %	Logistic EuroSCORE, %	Aortic valve gradient,mmHg	Aortic valve area
**Barth**	2012–2016	ACURATE neo	81 + 5	44.1	NA	18.8 ± 14.7	44 ± 15	0.68 ± 0.18
		SAPIEN 3	81 + 6	44.4	NA	19.1 ± 13.6	45 ± 14	0.67 ± 0.17
**Costa**	2014.09–2018.02	Evolut R	83(80–85)	29.2	3.9 ± 2.3	NA	52.8 ± 14.1	NA
		ACURATE neo	82(80–85)	31.2	4.0 ± 3.3	NA	51.3 ± 14.5	NA
		SAPIEN 3	83(82–85)	31.2	3.8 ± 1.7	NA	51.3 ± 17.2	NA
**Costa**	2017.09–2022.01	Evolut PRO	82(78–85)	46	3.4(2.3–4.7)	NA	44(35–53)	0.7(0.6–0.8)
		SAPIEN 3 Ultra	82(77–86)	46.1	3.1(2.1–4.9)	NA	44(36–51)	0.7(0.5–0.8)
**Finkelstein**	2012.02–2016.12	Evolut R	82(76–86)	61.9	3.2(2.2–4.7)	NA	40(33–53)	0.7(0.6–0.8)
		SAPIEN 3	82(78–85)	66.7	3.2(2.3–4.8)	NA	43(33–52)	0.7(0.6–0.8)
**Hase**	2013.10–2017.05	Evolut R	86(84–89)	15.9	6.4(4.8–8.4)	NA	49(35–69)	NA
		SAPIEN 3	87(82–89)	17.4	6.1(4.7–8.6)	NA	48(40–64)	NA
**Husser**	2014.01–2016.01	ACURATE neo	81 ± 6	39.2	NA	18 ± 10	45 ± 15	NA
		SAPIEN 3	81 ± 6	44.7	NA	18 ± 12	44 ± 16	NA
**Lanz/Kim**	2017.02–2019.02	ACURATE neo	82.6 ± 4.3	41	3.7(2.5–4.9)	NA	43 ± 17	0.7 ± 0.2
		SAPIEN 3	83.0 ± 3.9	45	3.4(2.6–5.2)	NA	42 ± 15	0.7 ± 0.2
**Mangieri**	2013.06–2018.10	Evolut R/PRO	79.1 ± 7.8	62.3	4.4 ± 3.1	NA	49.3 ± 16.5	NA
		SAPIEN 3	79.4 ± 7.9	53.3	4.2 ± 2.5	NA	49.4 ± 16.7	NA
**Mauri**	2014.02–2016.08	ACURATE neo	82.8 ± 6.5	7.6	NA	16.2 ± 8.8	46 ± 16	0.68 ± 0.19
		SAPIEN 3	81.9 ± 5.3	7.6	NA	16.6 ± 8.8	47 ± 16	0.65 ± 0.17
**Pellegrini**	2019.03–2021.12	ACURATE neo2	82(79–85)	49.4	NA	13.8(7.9–23)	43(34–52)	NA
		SAPIEN 3 Ultra	82(78–85)	47.9	NA	12.5(7.9–21.8)	42.5(34.8–51)	NA
**Potratz**	2014.06–2019.12	Evolut Pro	82.5 ± 5.1	42	NA	NA	49.1 ± 15.9	0.69 ± 0.17
		SAPIEN 3	82.9 ± 6.7	40	NA	NA	47.8 ± 18.1	0.69 ± 0.17
**Rheude**	2014.11–2020.12	Evolut R/PRO	82(78–85)	48.2	NA	12(7.8–20.3)	43(35–53)	NA
		SAPIEN 3 Ultra	82(77–85)	49	NA	11.9(7.6–21.5)	44(36–53)	NA
**Schaefer**	2012–2016	ACURATE neo	81.7 ± 5.5	28	5.8 ± 3.8	15.9 ± 9.3	NA	NA
		SAPIEN 3	81.2 ± 6.2	32	5.4 ± 3.6	13.7 ± 9.0	NA	NA
**Thiele/** **Feistritzer**	2016.04–2018.04	Evolut R	81.7 ± 5.3	47.9	4.9(2.9–9.9)	14.9(8.9–23.8)	38.5(30–50.5)	0.7(0.6–0.9)
		SAPIEN 3	81.5 ± 5.7	49.8	4.7(3.1–9.4)	14.8(8.6–24.4)	37(26.5–47.5)	0.8(0.6–0.9)
**Vlastra/** **van Nieuwkerk**	2010–2018	Evolut R	81.3 ± 7.1	43	6.6(4–12.8)	15(9.3–23.3)	51.1 ± 17.4	NA
		SAPIEN 3	81.5 ± 7.1	42	6.3(4–14.4)	15(9.7–23.0)	51.0 ± 17.6	NA

*STS* Society of Thoracic Surgeons

**Fig. 3:**
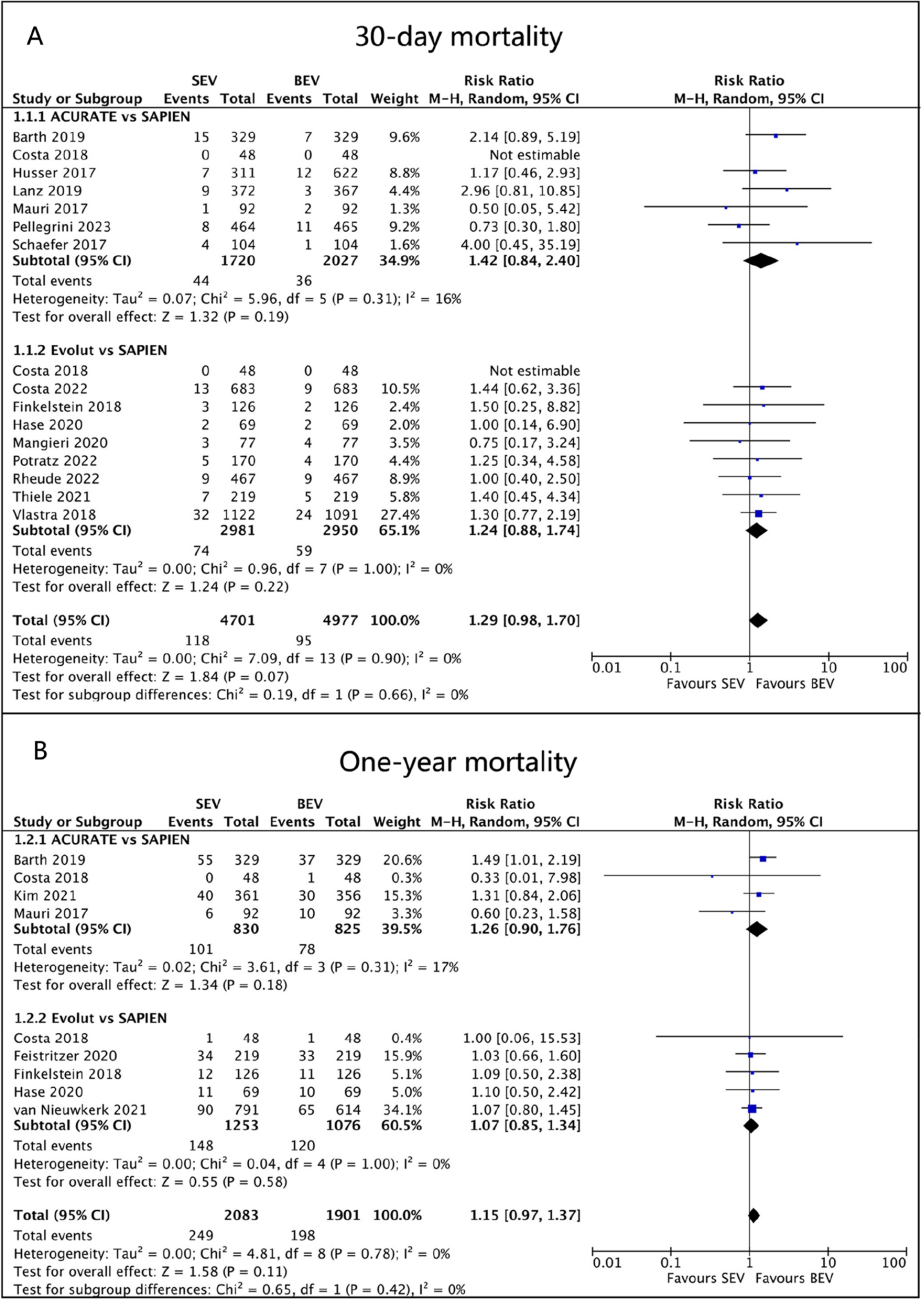
Forest plots. (**A**) 30-day mortality. (**B**) One-year mortality

Data from 15 studies were used to calculate the 30-day mortality rates. Among the 1720 patients treated with ACURATE SEV, 44 (2.6%) died, while 74/2981 (2.5%) deaths occurred in Evolut SEV and 95/4977 (1.9%) in SAPIEN BEV. SEV had a higher risk of death within 30 days compared to BEV in ACURATE (RR 1.42, 95% CI 0.84 to 2.40, *p* = 0.19, I^2^ = 16%) and Evolut (RR 1.24, 95% CI 0.88 to 1.74, *p* = 0.22, I^2^ = 0%), but the difference was not statistically significant (Fig. 3A). Data on one-year mortality were available from 8 studies. At the one-year follow-up, the risk of all-cause mortality was higher in the ACURATE (RR 1.26, 95% CI 0.90 to 1.76, *p* = 0.18, I^2^ = 17%) and Evolut (RR 1.07, 95% CI 0.85 to 1.34, *p* = 0.58, I^2^ = 0%) groups compared to BEV, but the difference was not statistically significant (Fig. 3B).

**Fig. 4:**
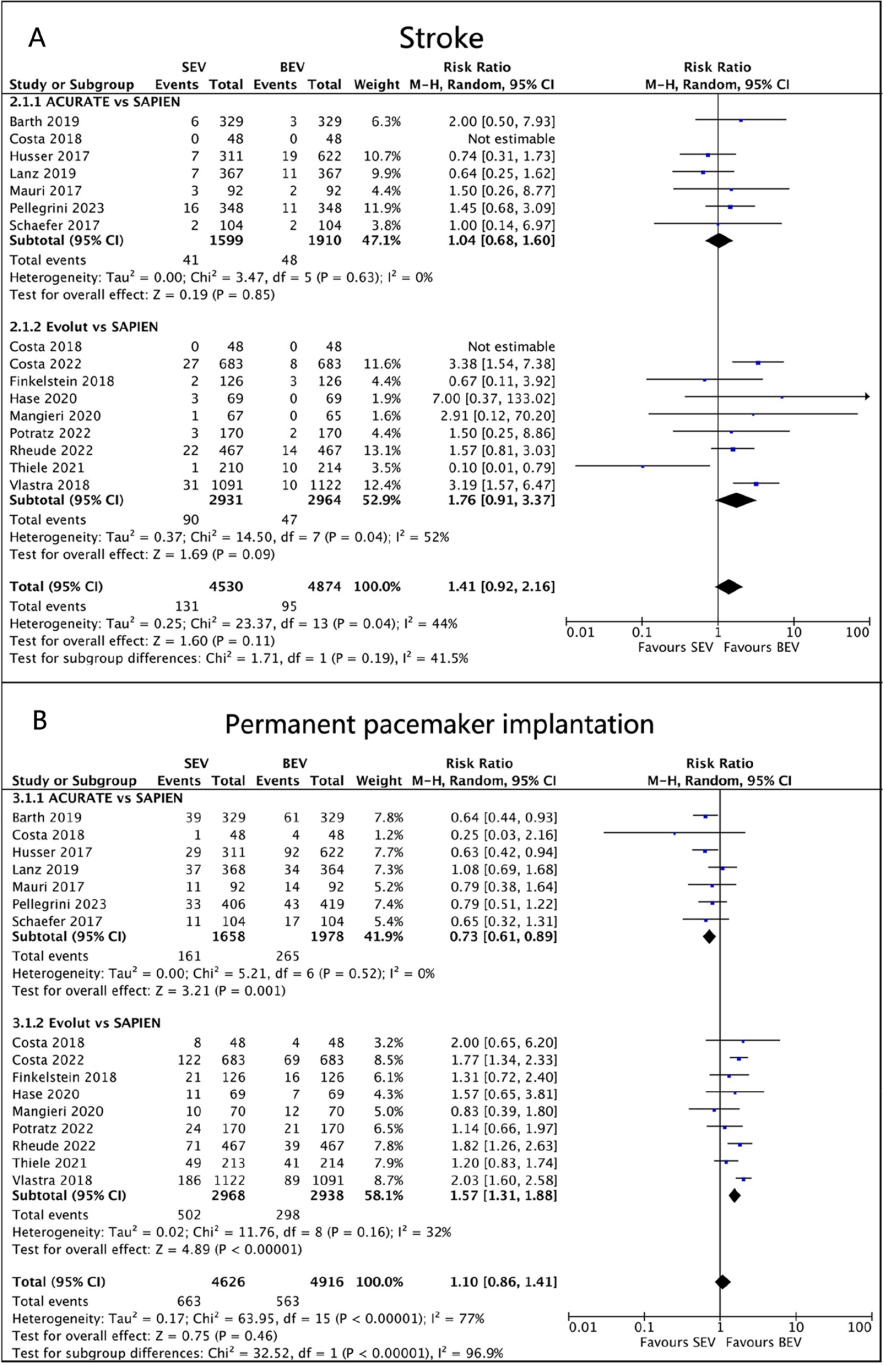
Forest plots. (**A**) Stroke. (**B**) Permanent pacemaker implantation

A total of 15 trials provided data on stroke incidence at 30 days. In the pooled analysis, ACURATE (RR 1.04, 95% CI 0.68 to 1.60, *p* = 0.85, I^2^ = 0%) and Evolut (RR 1.76, 95% CI 0.91 to 3.37, *p* = 0.09, I^2^ = 52%) were associated with an increased risk of stroke compared to SAPIEN, but the difference was not statistically significant (Fig. 4A). In a sensitivity analysis where the study by Thiele et al. was excluded, the use of Evolut SEV was significantly associated with a higher risk of stroke than the use of BEV (RR 2.31, 95% CI 1.57 to 3.39, *p* < 0.001, I^2^ = 0%). Subgroup analysis showed that SEV was associated with a higher risk of stroke in the PSM studies (RR 1.77, 95% CI 1.27 to 2.47, *p* = 0.0007, I^2^ = 10%).

Fifteen studies provided data on permanent pacemaker implantation (PPI). Among them, 161/1658 (9.7%) PPI patients were in ACURATE SEV, 502/2968 (16.9%) in Evolut SEV, and 563/4916 (11.5%) in SAPIEN BEV. Our analysis indicated that ACURATE had a lower risk of PPI compared to BEV (RR 0.73, 95%CI 0.61 to 0.89, *p* = 0.001, I^2^ = 0%). In contrast, Evolut had a relatively higher risk of PPI (RR 1.57, 95%CI 1.31 to 1.88, *p* < 0.00001, I^2^ = 32%; Fig. 4B).

**Fig. 5:**
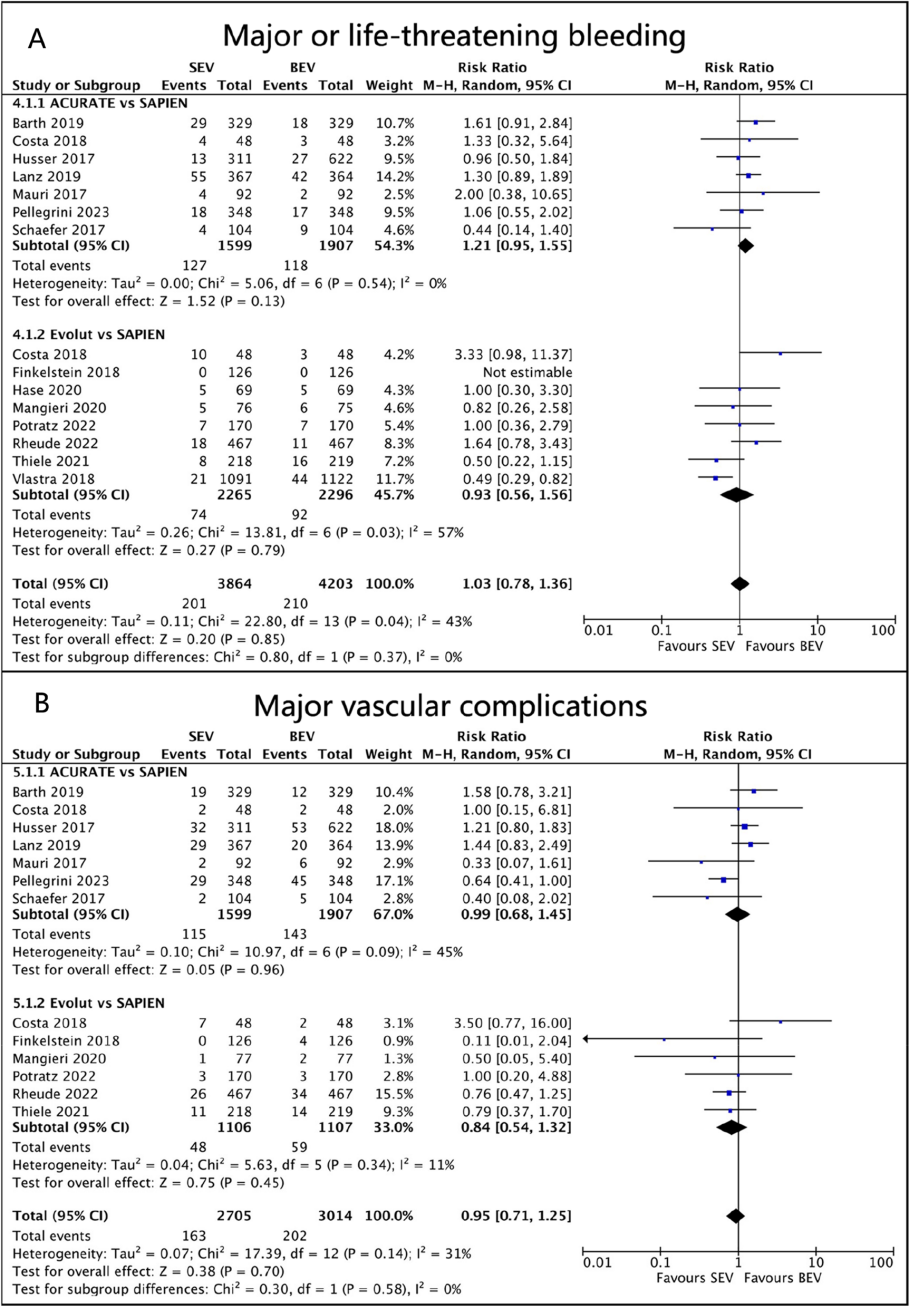
Forest plots. (**A**) Major or life-threatening bleeding. (**B**) major vascular complications

Data on major or life-threatening bleeding (MLTB) events were reported in 14 studies. Among 1599 patients receiving ACURATE SEV, 127 (7.9%) experienced bleeding, compared to 502/2968 (3.3%) with Evolut SEV and 210/4203 (5.0%) with SAPIEN BEV. Compared to BEV, ACURATE SEV was associated with a higher risk of bleeding (RR 1.21, 95% CI 0.95 to 1.55, *p* = 0.13, I^2^ = 0%), while Evolut SEV was linked to a lower risk (RR 0.93, 95% CI 0.56 to 1.56, *p* = 0.79, I^2^ = 57%). However, there was no significant difference between the groups (Fig. 5A).

The incidence of major vascular complications (MVC) was reported in 12 studies, and no significant difference was observed between SEV and BEV in the pooled results (ACURATE SEV: RR 0.99, 95%CI 0.68 to 1.45, *p* = 0.96, I^2^ = 45%; Evolut SEV: RR 0.84, 95%CI 0.54 to 1.32, *p* = 0.45, I^2^ = 11%; Fig. 5B). Specifically, there were 115/1599 (7.2%) MVC patients in ACURATE SEV, 48/1106 (4.3%) in Evolut SEV, and 188/2795 (6.7%) in SAPIEN BEV. In sensitivity analysis, after excluding the study by Pellegrini et al., the heterogeneity decreased, and there was still no statistically significant difference in the occurrence rate of MVC between ACURATE and SAPIEN (RR 1.21, 95%CI 0.89 to 1.65, *p* = 0.23, I^2^ = 7%).

**Fig. 6:**
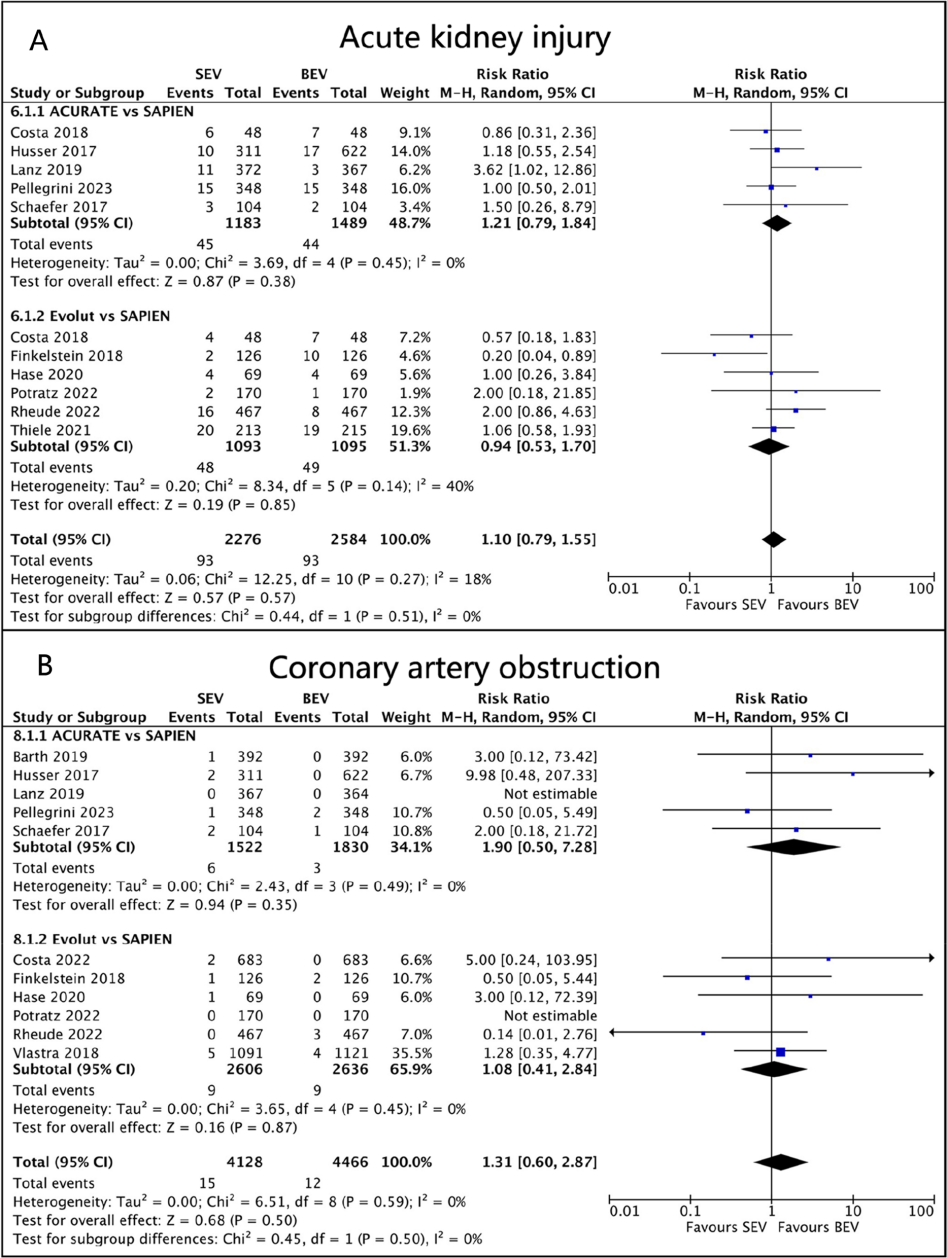
Forest plots. (**A**) Acute kidney injury. (**B**) Coronary artery obstruction

Data regarding acute kidney injury (AKI) were provided by 10 studies. There were 45/1183 (3.8%) AKI patients in ACURATE SEV, 48/1093 (4.4%) in Evolut SEV, and 93/2584 (3.6%) in SAPIEN BEV. There was no statistically significant difference between the groups (RR 1.21, 95%CI 0.79 to 1.84, *p* = 0.38, I^2^ = 0%; RR 0.94, 95%CI 0.53 to 1.70, *p* = 0.85, I^2^ = 40%; Fig. 6A**)**.

Results from eleven studies were included to assess the outcomes of coronary artery obstruction (CAO) following TAVR using ACURATE, Evolut, and SAPIEN valves. The incidence rates of CAO after valve implantation were 0.39%, 0.35%, and 0.27% for ACURATE, Evolut, and SAPIEN valves, respectively. SAPIEN BEV demonstrated a relatively lower risk of CAO compared to SEV, but no statistically significant differences were observed between SEV and BEV (RR 1.90, 95%CI 0.50 to 7.28, *p* = 0.35, I^2^ = 0%; RR 1.08, 95%CI 0.41 to 2.84, *p* = 0.87, I^2^ = 0%; Fig. 6B).

**Fig. 7:**
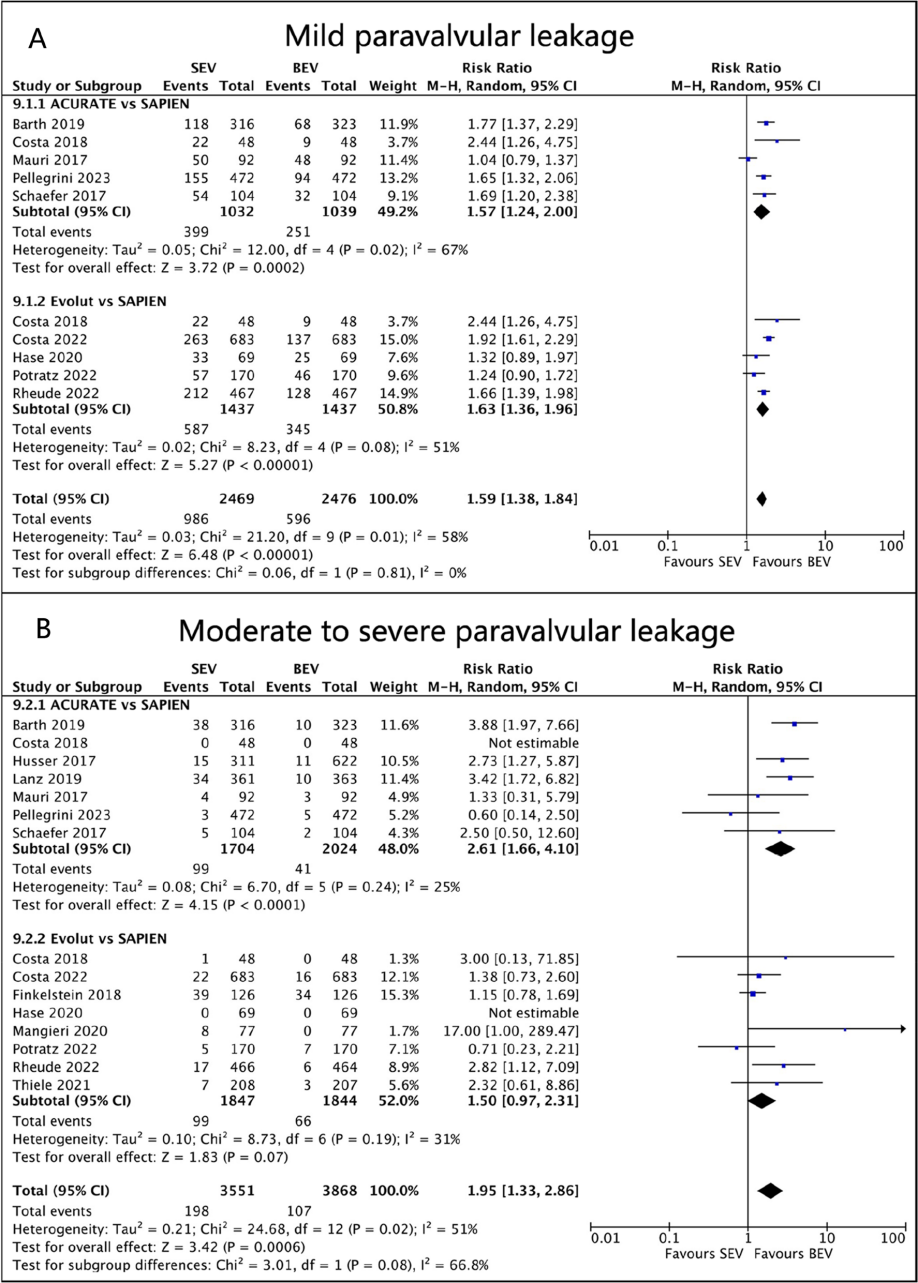
Forest plots. (**A**) Mild paravalvular leak. (**B**) Moderate to severe paravalvular leak

Eight studies reported the incidence of early mild paravalvular leak (PVL). There were 281/716 (39.2%) mild PVL patients in ACURATE SEV, 587/1437 (40.8%) in Evolut SEV, and 528/2153 (24.5%) in SAPIEN BEV. Compared to SAPIEN BEV, the incidence of mild PVL was higher in ACURATE SEV (RR 1.53, 95%CI 1.13 to 2.08, *p* = 0.007, I^2^ = 71%) and Evolut SEV (RR 1.63, 95%CI 1.36 to 1.96, *p* < 0.00001, I^2^ = 51%; Fig. 7A).

Data on the incidence of moderate to severe PVL were available in all 14 studies. There were 101/1717 (5.9%) moderate to severe PVL patients in ACURATE SEV, 99/1847 (5.4%) in Evolut SEV, and 107/3874 (2.8%) in SAPIEN BEV. The early incidence of moderate to severe PVL was significantly lower in SAPIEN BEV compared to ACURATE SEV (RR 2.62, 95%CI 1.65 to 4.14, *p* < 0.001, I^2^ = 27%) or Evolut SEV (RR 1.50, 95%CI 0.97 to 2.31, *p* = 0.07, I^2^ = 31%; Fig. 7B).

**Fig. 8:**
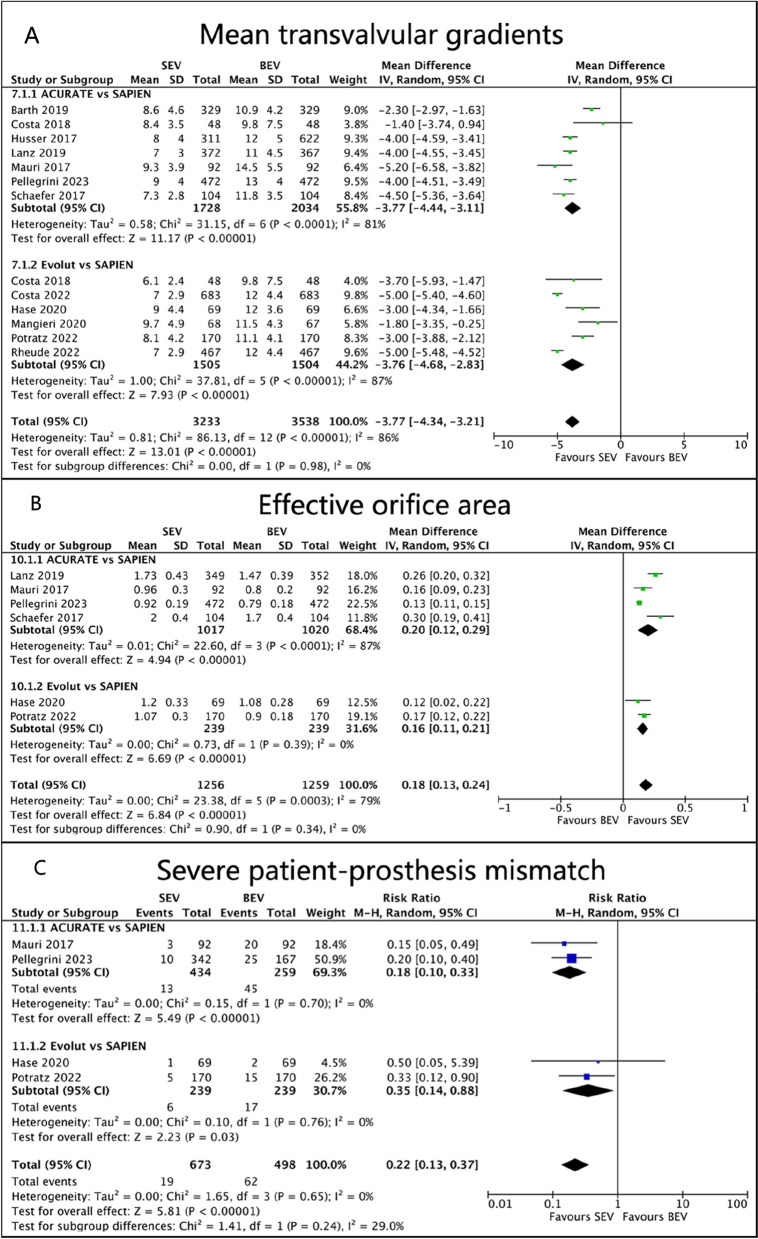
Forest plots. (**A**) Mean transvalvular gradients. (**B**) Effective orifice area. (**C**) Severe patient-prosthesis mismatch

Data on early mean transvalvular gradients (MTG) were provided by 12 studies. Our analysis showed that SAPIEN BEV had higher mean transvalvular gradients than ACURATE SEV (MD -3.77, 95%CI -4.44 to -3.11, *p* < 0.00001, I^2^ = 81%) and Evolut SEV (MD -3.76, 95%CI -4.68 to -2.83, *p* < 0.00001, I^2^ = 87%), but with high heterogeneity (Fig. 8A).

We included six studies in the analysis of effective orifice area (EOA). Our pooled analysis revealed that ACURATE SEV (MD 0.20, 95%CI 0.12 to 0.29, *p* < 0.00001, I^2^ = 87%) and Evolut SEV (MD 0.16, 95%CI 0.11 to 0.21, *p* < 0.00001, I^2^ = 0%) had a significantly larger EOA than SAPIEN BEV (Fig. 8B).

Only four studies provided data on the occurrence of severe patient-prosthesis mismatch (PPM) after TAVR. The results suggested that regardless of the type of SEV, the incidence of severe PPM was significantly lower than that of BEV (RR 0.18, 95%CI 0.10 to 0.33, *p* < 0.00001, I^2^ = 0%; RR 0.35, 95%CI 0.14 to 0.88, *p* = 0.03, I^2^ = 0%; Fig. 8C).

## Discussion

In the rapidly evolving field of TAVR technology, there are numerous THV options available, making the decision of which valve to use a daunting task. Presently, there are two primary types of valves: self-expanding valves (SEV) and balloon-expandable valves (BEV). The primary disadvantage of first-generation valves was the relatively large delivery system, which resulted in an increased incidence of vascular complications, a higher need for pacemakers, an elevated rate of paravalvular leak (PVL), and a greater incidence of stroke [30–33]. The new generation of valves aims to address these issues by reducing the outer diameter of the delivery catheter, providing repositionability and retrievability, enabling technical manipulation, and reducing TAVR-related complications (PVL, pacemaker implantation, and stroke). Prior studies have compared the two valves of the first generation [6, 7], but limited data exist comparing the new generation SEV with the BEV.

We conducted a meta-analysis comparing the postoperative outcomes of new generation SEV-TAVR and BEV-TAVR in RCT and PSM studies, including 4678 patients with SEV-TAVR and 4963 patients with BEV-TAVR. Our analysis yielded the following results: (1) No significant difference between SEV and BEV with regard to 30-day and 1-year mortality. (2) At 30-day, ACURATE SEV had a lower incidence of PPI compared to SAPIEN BEV, while Evolut SEV had a higher incidence of PPI compared to SAPIEN BEV. (3) There was no significant difference between the two valves regarding stroke, MLTB, MVC, AKI and CAO. (4) SEV had lower MTG and a larger EOA. (5) SEV was associated with a higher incidence of PVL.

Our study revealed that both ACURATE and Evolut SEV had higher mortality rates compared to SAPIEN valves after TAVR, but there was no significant difference in mortality between the two new generation valves. Generally, perioperative complications and baseline risk are associated with TAVR mortality. However, since our meta-analysis was conducted at the study level without patient-specific data, we cannot determine why SEV is associated with a higher death rate. Moderate to severe PVL is known to be associated with early and long-term mortality after TAVR in patients with aortic stenosis [34–36]. Our study found a significantly higher risk of PVL in SEV compared to BEV. Further studies are needed to verify the association between mild PVL and mortality in SEV.

Thirty-day stroke is a significant complication after TAVR, and its relationship with THV types remains unclear. Seppelt et al. reported stroke rates of 1.8% and 3.1% at thirty days after TAVR with SEV and BEV, respectively [37]. On the contrary, our research findings revealed that the early stroke incidence rates for Evolut SEV, ACURATE SEV, and SAPIEN BEV were 3.30%, 2.60%, and 1.90%, respectively. However, there was no statistically significant difference in the occurrence of stroke between SEV and BEV. Sensitivity analysis and subgroup analysis results indicate that the RCT conducted by Thiele et al. could be a potential source of heterogeneity [18]. They reported stroke rates of 0.5% and 4.7% for the Evolut group and SAPIEN group, respectively, with the stroke rate in the Evolut group significantly lower than the results of previous studies [24, 29]. Considering the numerous influencing factors, the differences in stroke between BEV and SEV should be cautiously interpreted. A possible explanation is that its larger device design, higher usage rate of balloon expansion, and repositionable and retrievable mechanism contribute to increased friction between the THV and the native aortic valve [29, 38, 39].

The incidence of stroke after TAVR is influenced by various factors. Grossman et al. found that the incidence of post-TAVR stroke varied among different medical centers [40]. As a result of enhanced diagnostic capabilities, comprehensive stroke center (CSC) sites exhibited a significantly higher in-hospital incidence of post-TAVR stroke compared to non-CSC sites (CSC: 2.65% vs. non-CSC: 1.15%, *p* < 0.001). On the other hand, the use of cerebral protection devices (CEP) may potentially reduce the incidence of postoperative stroke, but its efficacy has been a subject of debate. A large-scale PROTECTED TAVR RCT reported no significant difference in the occurrence of stroke within 72 h or before discharge between the CEP group and the control group (2.3% vs. 2.9%, *P* = 0.30) [41]. A recent meta-analysis by Wolfrum et al., incorporating four RCT and one PSM study, reported that the use of CEP can reduce the risk of overall stroke and disabling stroke, but it does not decrease the risk of non-disabling stroke [42]. Further large-scale clinical trials may provide more evidence on the effectiveness of CEP in TAVR procedures.

With the use of new-generation devices, the safety and efficacy of TAVR have significantly improved. However, compared to surgical replacement, permanent pacemaker implantation (PPI) remains a common post-procedure complication [43]. Previous studies evaluating TAVR patients with first generation valves showed higher rates of conduction disturbances and PPI in patients receiving the SEV compared with BEV [44–46]. It is noteworthy that our study demonstrated a significant difference in the PPI rate between ACURATE and Evolut, two different SEV devices. Conduction disturbances after valve implantation are primarily due to contact between the prosthetic valve and the left ventricular outflow tract. The depth of valve implantation may be an important influencing factor for post-TAVR PPI. Abdelfattah et al.'s meta-analysis suggested that greater depth of valve implantation serves as a predictive factor for early PPI and left bundle branch block after TAVR [47]. Similarly, a retrospective study encompassing 1,028 TAVR patients implemented a high-deployment technique (HDT) in 406 patients. The HDT group exhibited decreased rates of 30-day PPI (5.5% vs. 13.1%; *P* < 0.001) [48].

Our research findings revealed that the early PPI rates for ACURATE SEV, Evolut SEV, and SAPIEN BEV were 9.70%, 16.9%, and 11.5%, respectively. The lower risk of PPI associated with ACURATE SEV may be attributed to its large-cell design, resulting in lower radial forces, as well as its top-down deployment mechanism, which minimizes interference with the left ventricular outflow tract during expansion [49]. On the contrary, in Evolut R/PRO, a higher incidence of PPI was observed compared to ACURATE and SAPIEN, possibly due to its frame design, which makes it more susceptible to further protrusion into the left ventricular outflow tract [50]. Nonetheless, through the implementation of Cusp-Overlap technology and new-generation delivery systems, the incidence of PPI with Evolut SEV has experienced a significant decrease.

The CHOICE randomized clinical trial [6] reported the incidence of early major vascular complications of first generation SEV versus BEV (9.9% vs 11.1%). New generation valves focus on improving device technology by modifying valve design and miniaturizing delivery systems, allowing safe use in a wider range of patients and reducing vascular injury [51, 52]. Our meta-analysis found comparable risks of early MVC for SEV and BEV (6.0% vs 6.7%). Because the risk of vascular complications should theoretically depend only on local anatomy and surgical technique [53]. Similarly, our data showed no significant difference between the new generation SEV and BEV regarding the risk of MLTB, AKI, and a decreased incidence compared with the first generation valves.

Coronary artery obstruction is a rare but devastating complication of TAVR. Our study demonstrated a lower incidence of CAO with BEV compared to SEV, but without statistical significance. Ochiai et al. reported the prevalence of CT-defined post-TAVR coronary access unfavorable features when using BEV and SEV (Evolut R/PRO: left coronary artery/right coronary artery = 34.8/25.8%; SAPIEN 3: left coronary artery/right coronary artery = 15.7/8.1%) [54]. Unfortunately, the studies included in our analysis did not report the occurrence of postoperative coronary access unfavorable features. TAVR may pose challenges to future coronary access and aortic valve re-interventions in a large cohort of low-risk patients [55]. SAPIEN BEV, with a lower stent frame height, large cells design, and an intra-annular valve design, typically allows coronary re-intervention above the valve's outflow tract or through the large cells of the frame. The stent frame height of Evolut SEV extends to the coronary artery ostium with a supra-annular valve design, making coronary re-intervention feasible only through the diamond-shaped cells after valve deployment. ACURATE SEV's design with large cells facilitates the passage of guidewires and catheters but sacrifices some radial support strength of the device, making it more challenging in patients with heavily calcified leaflets and potentially increasing the risk of PVL.

Overall, BEV may be more suitable for patients at a higher risk of CAO and who are younger and may require future interventions. The application of coronary protection techniques, such as chimney stenting or BALISICA (Bioprosthetic or native Aortic Scallop Intentional Laceration to prevent Iatrogenic Coronary Artery obstruction during TAVR) technique, can effectively prevent the occurrence of CAO [56]. If SEV valves are chosen, the method of commissural alignment can reduce the occurrence of post-implantation CAO and optimize coronary access, thereby increasing options for future interventions [57, 58].

Previous studies have demonstrated differences in hemodynamic performance between first generation SEV and BEV. The CoreValve SEV, when compared to the SAPIEN BEV, was found to have a significantly lower residual gradient [59]. Both types of new generation valves have shown improved hemodynamic performance. However, our study suggests that the new generation SEV has superior antegrade hemodynamic performance when compared to the new generation BEV. This difference is likely due to the supra-annular position of the SEV leaflets, which allows for lower resistance to left ventricular outflow and gradients [60, 61]. Our study also pooled data on effective orifice area (EOA) or effective orifice area index (EOAi), an indicator that defines patient-prosthesis mismatch (PPM) in patients after TAVR. Prior studies have suggested that PPM is more common in BEV than SEV, while some studies have shown an increase in PPM in the new generation valves compared to the first generation valves [62–64]. However, with the introduction of skirt designs in most new generation valves, particularly double skirt designs [62, 65], the EOA is reduced to a certain extent. We found that the new generation SEV has a larger EOA and lower mean pressure gradients than the new generation BEV. Hemodynamic performance may be the primary cause of structural valve dysfunction (SVD). A retrospective analysis of 300 TAVR patients with first generation valves by Deutsch et al. demonstrated a significant difference in the incidence of SVD between SEV and BEV (CoreValve 11.8% vs SAPIEN 22.6%, *p* = 0.01) [66]. The CHOICE RCT found that the incidence of SVD was higher in SEV than in BEV at 5-year follow-up (6.6% vs 0.0%, *p* = 0.02). Due to a lack of data, we were unable to compare the incidence of SVD in the new generation valves. Longer follow-up studies are needed to investigate the differences in SVD between the new generation SEV and BEV.

PVL can have a negative impact on clinical outcomes and can even negate the survival benefit of TAVI in patients with moderate or severe PVL [67]. A previous meta-analysis reported that 11.7% of patients treated with first generation valves developed moderate to severe PVL [68]. New generation valves are generally designed to be repositionable and provide a better seal with the native valve, thereby reducing the chance of PVL occurring between the prosthesis and annulus. Kowalewski et al. showed that the incidence of moderate or severe PVL was lower in Evolut R SEV compared to CoreValve SEV (RR 0.55, 95%CI 0.39 to 0.79, *p* = 0.01) [69]. According to a meta-analysis by Ando et al., SAPIEN 3 BEV showed a decrease from 6.9% to 1.6% in moderate and severe PVL [70]. In our study, 5.6% and 2.7% of new-generation SEV and BEV, respectively, had moderate to severe PVL. The lower risk of moderate to severe PVL with BEV may be due to its higher radial force and better adaptation to the aortic valve annulus. Mild PVL was present in 40.3% and 24.5% of BEV and SEV cases, respectively, and further studies are needed to investigate its effect on postoperative outcomes.

Our study has some limitations, the most significant being the predominant inclusion of observational studies in our literature review. Despite incorporating propensity score matching studies to ensure comparable baseline characteristics, they are limited by potential flaws and unidentified biases. Moreover, we observed a high degree of heterogeneity in some of the outcomes. The central valve preference may be an easily overlooked factor when comparing self-expanding and balloon-expanding valves, as shown in a study by Witberg et al., which found worse perioperative outcomes, moderate to severe aortic regurgitation, and 2-year mortality when TAVR was performed with SEV in BEV-dominant centers [71]. Therefore, subgroup analysis based on central valve preference is necessary. Another limitation is that the maximum follow-up period for our study was 1 year, and data on valve durability and long-term outcomes are still required. Additionally, our analysis found differences in postoperative outcomes between different SEV, but we did not conduct a comparison between different SEV. Valve selection can be individualized according to patient characteristics or anatomy, and using a specific valve type in a given patient may lead to better outcomes. A meta-analysis based on the clinical characteristics of the patients would have provided stronger evidence.

## Conclusion

In terms of 30-day mortality, stroke, bleeding, MVC, AKI, CAO, and one-year mortality, there was comparability between the two valve types following TAVR. SEV was associated with better hemodynamic outcomes, except for a higher incidence of PVL. Compared to SAPIEN BEV, ACURATE SEV had a lower risk of PPI at 30 days, while Evolut SEV was associated with a higher risk of PPI. These findings underscore the importance of personalized valve selection.

## Supplementary Information

**Additional file 1. **

## Data Availability

All data generated or analyzed during this study are included in this manuscript and its additional files.
