# Supplementary material for: Comparison of outcomes of self-expanding versus balloon-expandable valves for transcatheter aortic valve replacement: a meta-analysis of randomized and propensity-matched studies
Source: BMC Cardiovasc Disord. 2023 Jul 31;23:382. doi: 10.1186/s12872-023-03397-3 (PMC10388567; doi:10.1186/s12872-023-03397-3)

**Supplementary Table 1**. Database search strategy

**Supplementary Table 2:** Study quality of included comparative observational studies using the Newcastle-Ottawa scale.

| **Author/Study** | **Year** | **Study quality (Newcastle-Ottawa Scale)** | | | |
| --- | --- | --- | --- | --- | --- |
|  |  | Selection | Comparability | Outcome/exposure | Total score |
| Barth | 2019 | ******** | ****** | ******* | 9 |
| Costa | 2018 | ******** | ****** | ******* | 9 |
| Costa | 2022 | ******** | ****** | ******* | 9 |
| Finkelstein | 2018 | ******** | ****** | ****** | 8 |
| Hase | 2020 | ******** | ****** | ******* | 9 |
| Husser | 2017 | ******** | ****** | ****** | 8 |
| Mangieri | 2020 | ******** | ****** | ******* | 9 |
| Mauri | 2017 | ******** | ****** | ******* | 9 |
| Pellegrini | 2023 | ******** | ****** | ******* | 9 |
| Potratz | 2022 | ******** | ****** | ******* | 9 |
| Rheude | 2022 | ******** | ****** | ******* | 9 |
| Schaefer | 2017 | ******** | ****** | ****** | 8 |
| van Nieuwkerk | 2021 | ******** | ****** | ****** | 8 |
| Vlastra | 2018 | ******** | ****** | ****** | 8 |

**Supplementary Table 3:** Risk of bias of included randomized controlled trials.

| **Trial** | **Year** | **Random sequence generation (selection bias)** | **Allocation concealment (selection bias)** | **Blinding of participants and personnel (performance bias)** | **Blinding of outcome assessment (detection bias)** | **Incomplete outcome data (attrition bias)** | **Selective reporting (reporting bias)** | **Other bias** |
| --- | --- | --- | --- | --- | --- | --- | --- | --- |
| Lanz | 2019 | Low risk | Low risk | Unclear risk | Low risk | Low risk | Low risk | Low risk |
| Kim | 2021 | Low risk | Low risk | Unclear risk | Low risk | Low risk | Low risk | Low risk |
| Thiele | 2020 | Low risk | Low risk | Unclear risk | Low risk | Low risk | Low risk | Low risk |
| Feistritzer | 2021 | Low risk | Low risk | Unclear risk | Low risk | Low risk | Low risk | Low risk |

**Supplementary Figure 1.** Subgroup analysis based on the study's design type.


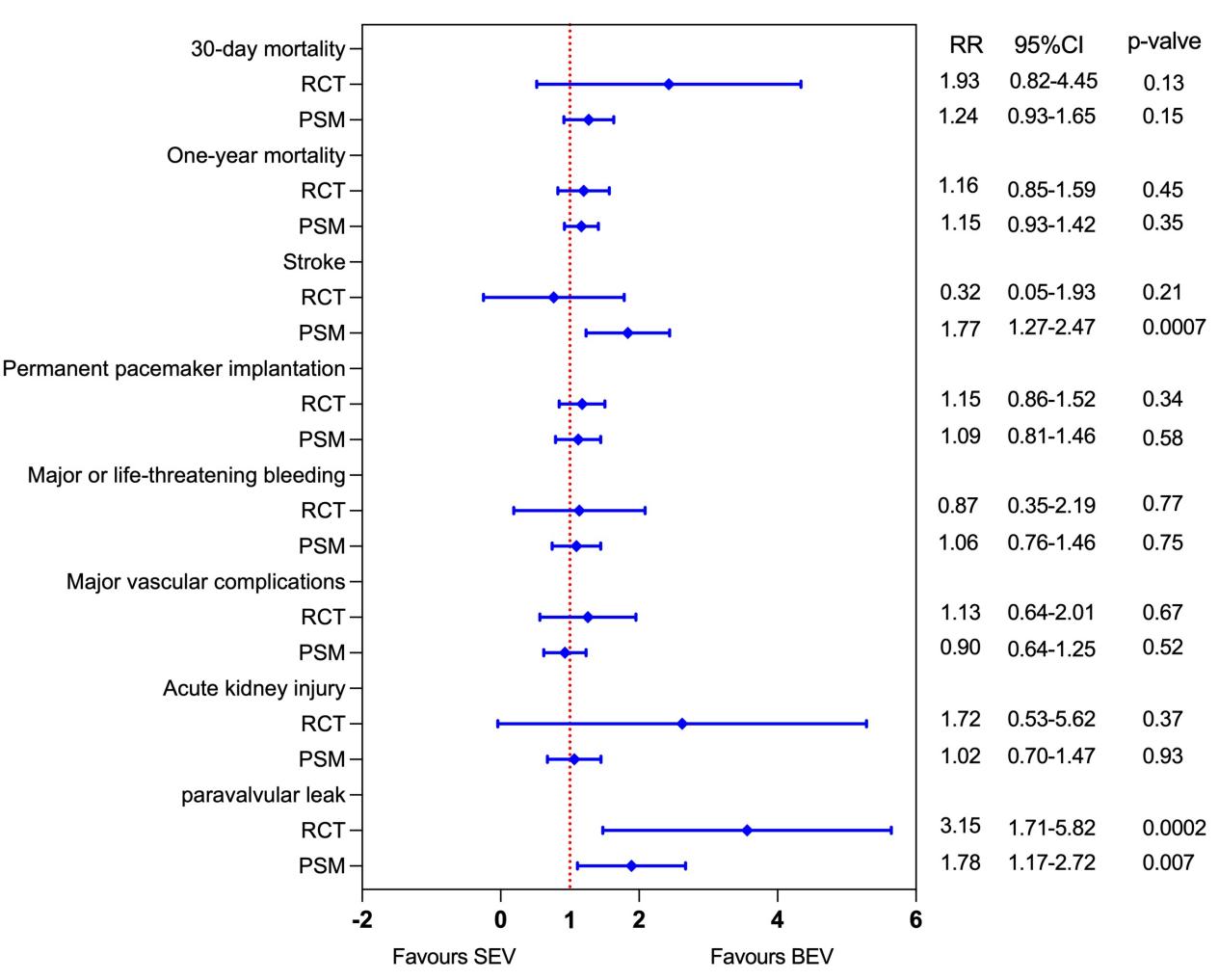


**Supplementary Figure 2.** Funnel plots


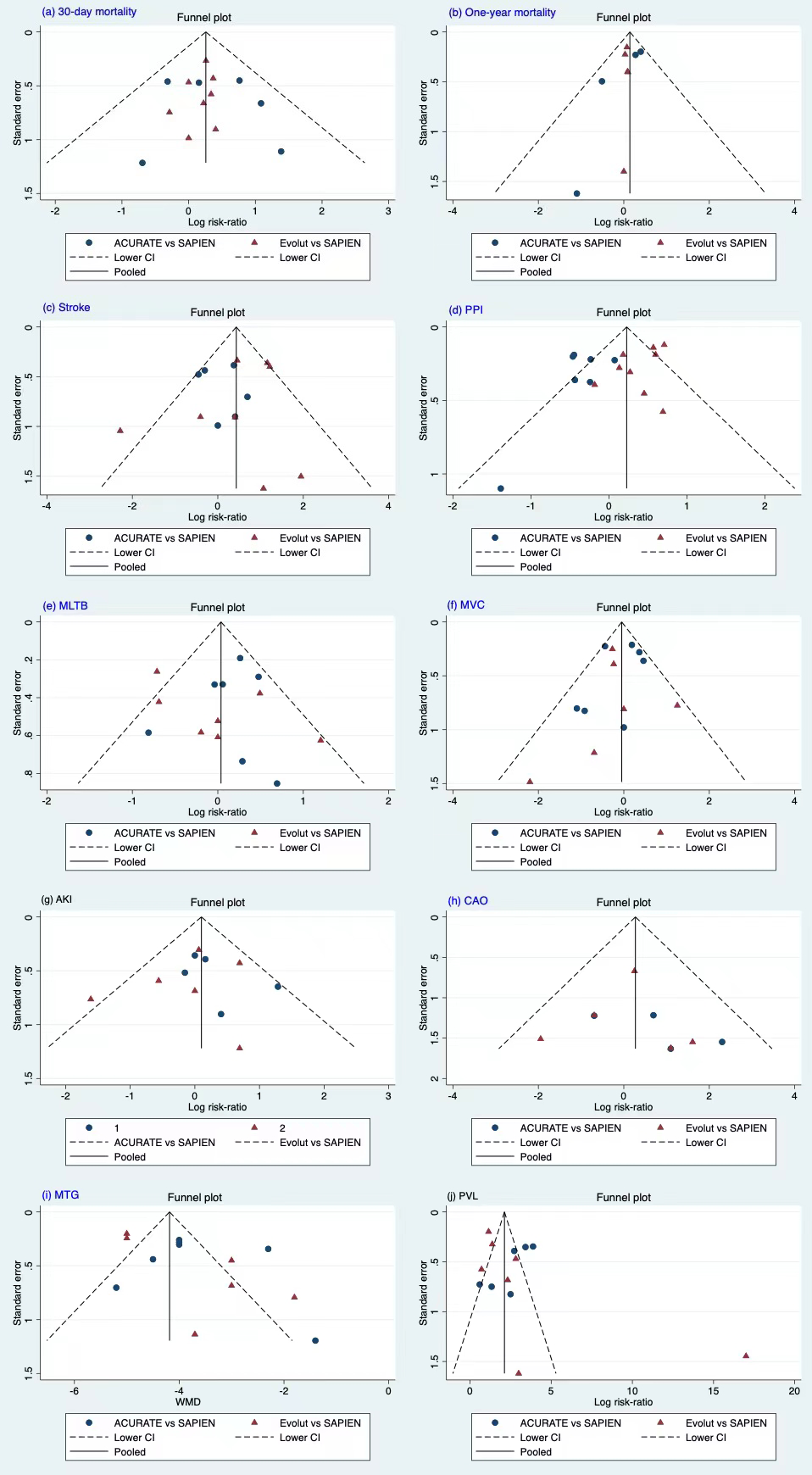

Supplement: Supplementary file 1 — Additional file 1. [file 12872_2023_3397_MOESM1_ESM.docx]
